# Perspective: Reducing SARS-CoV2 Infectivity and Its Associated Immunopathology

**DOI:** 10.3389/fimmu.2020.581076

**Published:** 2020-10-22

**Authors:** Abhishek Dubey, Surbhi Dahiya, Barry T. Rouse, Sharvan Sehrawat

**Affiliations:** ^1^Department of Biological Sciences, Indian Institute of Science Education and Research Mohali, Mohali, India; ^2^Biomedical and Diagnostic Sciences, College of Veterinary Medicine, The University of Tennessee, Knoxville, TN, United States

**Keywords:** inflammation, therapy, sdAb, immunoregulation, viruses

## Abstract

COVID-19 has become difficult to contain in our interconnected world. In this article, we discuss some approaches that could reduce the consequences of COVID-19. We elaborate upon the utility of camelid single-domain antibodies (sdAbs), also referred to as nanobodies, which are naturally poised to neutralize viruses without enhancing its infectivity. Smaller sized sdAbs can be easily selected using microbes or the subcellular organelle display methods and can neutralize SARS-CoV2 infectivity. We also discuss issues related to their production using scalable platforms. The favorable outcome of the infection is evident in patients when the inflammatory response is adequately curtailed. Therefore, we discuss approaches to mitigate hyperinflammatory reactions initiated by SARS-CoV2 but orchestrated by immune mediators.

## Introduction

Severe acute respiratory syndrome coronavirus-2 (SARS-CoV2) caused COVID-19 pandemic has impacted almost all countries, and it has caused a widespread shutdown in normal lifestyle worldwide (1). Control measures are ongoing, and some countries have seemingly achieved reasonable success in either managing the spread of the disease or reducing its severity in patients. The efforts are underway to unravel details on COVID-19 epidemiology, pathogenesis and more importantly to expeditiously generate modalities to reduce its consequences. The human to human transmission of SARS-CoV2 occurs *via* respiratory droplets or probably through aerosols (2). The droplets settle on surfaces and can retain some infectivity for several days (3). The transmission of the virus through contaminated surfaces followed by a potent infection in humans is not unequivocally established (4). Whether or not the virus remains associated with epithelial or stromal cells, sloughed off from the mucosae of the inflamed buccal or nasal cavity, is unknown at present but has been shown for some other respiratory tract pathogens (5). Even though the presence of SARS-CoV2 in the exosomes has not been analyzed, the genomes of some viruses, such as that of hepatitis C virus, have been previously found in the exosomes (6). The transmission of SARS-CoV2 from mother to fetus in utero is also not firmly established but seems unlikely (7). This could be because of the observed limited episodes of lower intensity viremia in infected individuals (8).

COVID-19 has immunopathological manifestations and therefore the optimal control of inflammatory response could result in a favorable outcome (9–11). The category of immunosuppressants that should be used in COVID-19 patients is debated, as such interventions invariably have long term ill-effects likely to be more pronounced in aged individuals because of their hematopoietic inefficiencies (12). Most epidemics or pandemics can be conveniently controlled if effective vaccines are produced and be made available for the susceptible populations. Therefore, elaborate efforts are being made to develop vaccines against SARS-CoV2. Many such vaccine candidates are already in their Phase II/III of clinical trials (A complete list of COVID-19 vaccines currently under trial can be found at https://www.who.int/publications/m/item/draft-landscape-of-covid-19-candidate-vaccines). Curiously, by the time vaccines against the circulating coronaviruses were produced earlier, the epidemics were over and the vaccines were never properly evaluated for safety, efficacy, and longevity of protection. In this communication, we focus on approaches that are likely to be valuable in the near future as the development of vaccines and assessment of their safety and efficacy is a lengthy process. Furthermore, vaccines against some animal coronaviruses, such as the one causing feline infectious peritonitis in cats, have been less effective (13, 14). Contrarily, some vaccines against the SARS viruses enhanced disease severity when tested in a mouse model (14). The underlying mechanisms for enhanced infectivity are still poorly understood but could involve an antibody-dependent enhancement (ADE) of the infection followed by immunopathologies (15). Poorly neutralizing antibodies or their low abundance could fail to efficiently neutralize the virus. Such antibodies nonetheless interact with the exposed viral antigens to enhance the infectivity as well as broaden the cellular tropism (16). SARS-CoV2 reactive antibodies and T cells against epitopes on different proteins of SARS-CoV2 have been reported in apparently healthy individuals (17–21). Therefore, the effects of pre-existing immunity against coronaviruses in the pathogenesis of COVID-19 needs to be better studied.

We discuss two main lines of interventions, some of which have already been tried or should be extensively investigated to minimize the impact of the disease. One such intervention is to limit the success of the virus entry by blocking interactions of the viral proteins with the primary cellular entry receptor, i.e., angiotensin-converting enzyme 2 (ACE2). Several approaches have been explored for the previously circulating SARS coronaviruses, including SARS-CoV and Middle East Respiratory Syndrome-coronavirus (MERS-CoV) (22–24). An approach that we favor and elaborate upon, which has received limited evaluation, involves the use of sdAbs (also called as nanobodies) that can be generated from the genetic scaffolds of rearranged heavy chain only antibodies (HCAbs), known to occur in all camelids (25). Though sdAbs can be generated from the genetic fragments of conventional antibody having four chains, the term sdAbs used in the article refers to those of camelid origin. Such sdAbs are aptly suited to neutralize pathogens, toxins, and inflammatory mediators (26–29). With the increasing number of studies evaluating B and T cell responses against different epitopes in SARS-CoV2, sdAbs can be selected against multiple viral epitopes to yield potent, multi-specific formulations. Furthermore, sdAbs against host proteins such as MHC class II molecules can additionally be engineered to deliver viral antigens or peptides to the antigen-presenting cells (APCs) to enhance the efficiency of cross-presentation pathways in order to generate potent anti-viral CD8^+^ T cell response (30).

A further approach that merits more evaluation is the optimal management of inflammatory response to SARS-CoV2 as its resolution favors survival over lethality (31, 32). Accumulating evidence demonstrates that COVID-19 is predominantly an immunopathological response resulting from dysregulated differentiation of innate and adaptive immune cells following SARS-CoV2 infection (10, 11, 33).We highlight some anti-inflammatory approaches that can help mitigate inflammation to achieve a favorable disease outcome.

## Factors Influencing the Disease Susceptibility and the Outcome of SARS-CoV2 Infection

The immune status of host at the time of infection, exposure history, co-morbidities, age at which infection occurs and the dose as well as the formulation of inoculum could influence the disease outcome (34, 35). Some of the SARS-CoV2 infected children exhibited heightened inflammatory response, but the overall susceptibility and severity of COVID-19 in this age group are rare (36). This might relate to their lower ACE2 expression levels, potent type I IFN response or the cross-protection offered by routinely used vaccines (36–38). Aged as well as the young individuals with one or more comorbidities, such as autoimmune disorders, cardiovascular, pulmonary or renal insufficiencies are more likely to develop severe COVID-19 infections. Accordingly, lethal disease is ~100 times more prevalent in individuals above the age of 80 years as compared to those below 50 years of age (39, 40). The declining precursor frequencies of adaptive immune cells or their aberrant activation are commonly observed in aged individuals, but the functionality of innate immune cells in the aging population is less well explored (41, 42). Such an analysis could help explain their enhanced susceptibility to viral infections and might additionally suggest control measures. The SARS-CoV2 entry receptor, ACE2, is highly expressed in most of the critical organs such as lungs, heart, kidneys, and the endothelial lining of blood vessels in the central nervous system (CNS) (43). Therefore, these anatomical locations could serve as the virus predilection sites. Pathogens that replicate rapidly in the host and infect critical organs could outrun innate immune mechanisms such as the activity of NK cells or type I IFN pathways (44, 45). Coronaviruses are known to disable one or the other components of type I IFN pathways and can spread unabated within the host in early stages (46). It is conceivable that the initial viral load could critically influence the disease outcome by tipping the balance toward either a predominantly immunopathological event or the one that resolves with mild or subclinical manifestations. Therefore, appropriate management of COVID-19 cases should aim to control the initial viral loads by promoting the endogenous anti-viral mechanisms such as the type I IFN pathways or the activity of NK cells (47, 48). The exogenously supplied type I IFNs or the viral neutralizing antibodies could also control the viral burden (49–51). Type I IFNs have been associated with the severe COVID-19 cases, therefore their contribution in the pathogenesis warrants further investigation (49). The high viral loads induce hyperinflammatory reactions commonly referred to as cytokine storm (10, 11, 33). The enhanced levels of cytokines and other inflammatory molecules such as IL-6, IL1β, IL-8, IL-17, MCP1, MIP1α, C-reactive proteins were observed in COVID-19 patients (10, 11, 33). An induction of robust CD4+ T cell response and an exhaustion of CD8+ T cells have been suggested to occur in severe cases of COVID-19 (52). SARS-CoV2 causes an acute infection followed by the virus control in most individuals. Therefore, the functional consequences of the expressed markers associated with the exhaustion phenotype in immune cells and are commonly observed during chronic infections can be debated upon in the pathogenesis of COVID-19. The immunoregulatory response suppresses the initiation of inflammation. One of the major regulatory T cells (Tregs) express Foxp3 transcription factor. However, such cells could acquire proinflammatory roles when home to the inflammatory sites because of their plasticity (53). Therefore, approaches that could promote endogenous Treg responses or stabilize their phenotype could help dampen the inflammation (54). Such approaches could be more valuable when the virus is adequately controlled. We highlight some such strategies in a subsequent section.

## Coronavirus Entry and Replication Events Provide Insights Into Potential Anti-Viral Targets

Coronaviruses are positive-sense, single-stranded RNA viruses of the Coronaviridae family, with approximately two-thirds of the genomes encoding for the non-structural proteins (NSPs) (55). The viral structural proteins and the host-derived lipid bilayers make the outer surface of the virus. Coronaviruses primarily consist of four structural and accessory proteins, i.e., Spike (S), Envelope (E), Membrane (M) and Nucleocapsid (N) proteins. Some β-coronaviruses also have hemagglutinin-esterase (HE) that facilitates virus binding to the host cell displayed sialic acid moieties (55). Binding of SARS-CoV2 to the cells requires the interaction of its homotrimeric S protein with cellular ACE2 (43, 56, 57) (**Figure S1**). Such interactions are also observed for SARS-CoV, but might occur with higher affinity for SARS-CoV2 (58). Clathrin-mediated endocytosis of coronaviruses is followed by the cleavage of its S protein by cellular serine proteases. This process exposes the fusion peptide (FP) which is responsible for viral fusion with the endocytic membranes (56). Subsequently, the viral genome is released into cytoplasm, subgenomic viral mRNAs are produced and translated into different structural proteins (55). Polyproteins (pp)1a and 1ab, encoded by ORFs rep1a and rep1b, are subsequently cleaved by the virus-encoded papain-like proteases (PLpro) and Main proteases (Mpro) to generate NSPs. The packaged viral genome is assembled to form new virions that are then exocytosed to initiate further rounds of infection. Many events and the host factors involved in the viral entry, trafficking and viral replication still remain poorly understood. For a detailed analysis of such events, some excellent studies and reviews should be consulted (55, 59). The RNA-dependent RNA polymerase lacks in the proofreading activity and therefore generates frequent mutations in the viral genome. Viral mutants thus generated could exhibit enhanced tissue tropism and affinity for different host proteins. An S protein mutant of SARS-CoV2 (D614G) was recovered from many COVID-19 patients worldwide, but detailed analysis on its infectivity and pathogenesis are currently lacking (60). Since the virus entry in susceptible cells is initiated by the interaction of viral S protein and the cellular ACE2 receptor, this step is considered as the prime target for anti-viral maneuvers.

**Figure 1:**
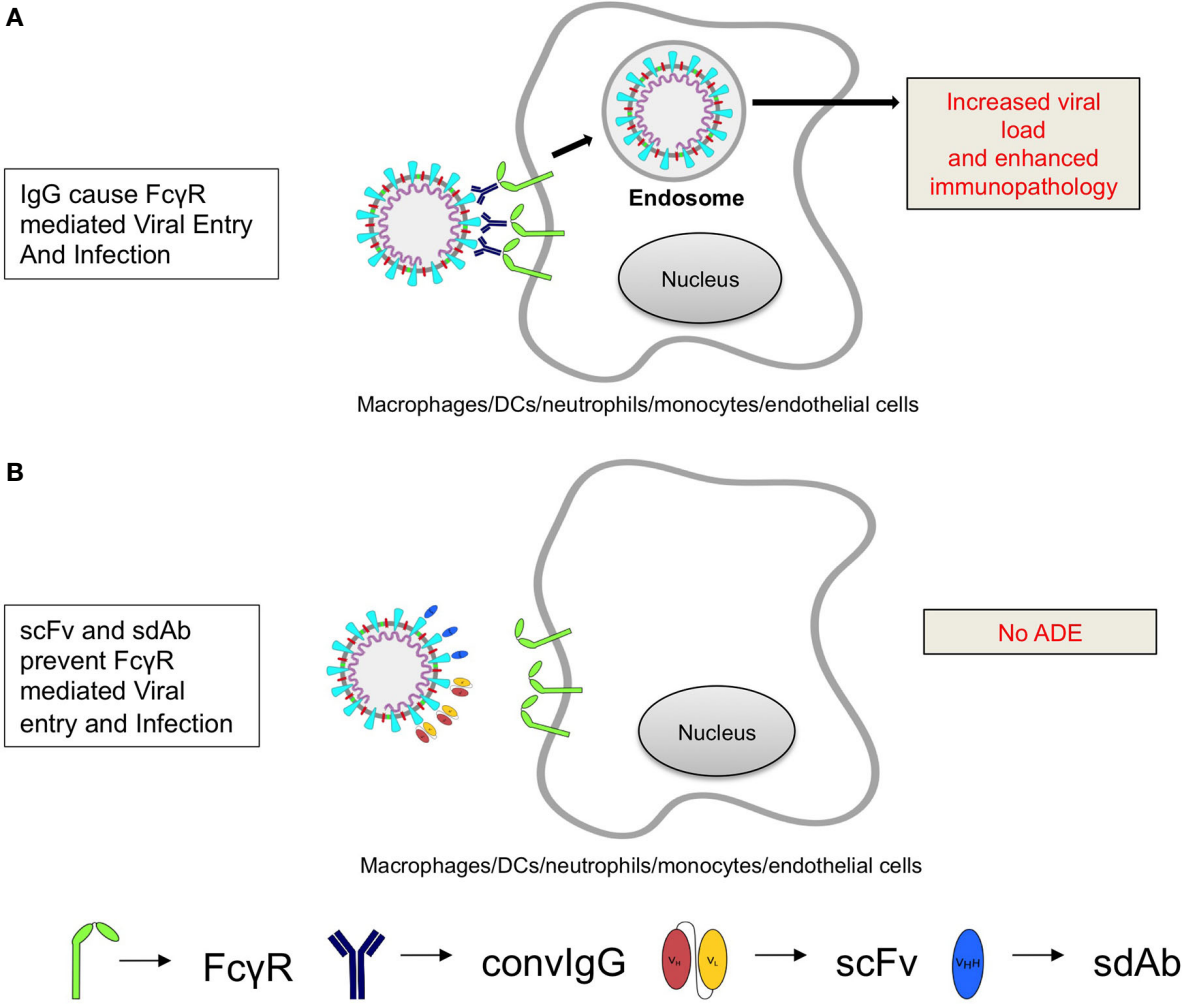
A cartoon shows the possible mechanisms of antibody dependent enhancement of SARS-CoV2 by poorly neutralizing antibodies. **(A)** Poorly neutralizing IgG antibodies bind to the viral epitopes *via* their Fab regions and ligate Fc receptors (FcγR) expressed by multiple cell types. The recycling of receptors in the cells promotes viral infection to increase viral load and that inturn promotes cytokine production to aggravate immunopathological response. **(B)** The smaller sized virus-specific antibodies (sdAbs or scFv that lack Fc region) efficiently neutralize the virus without causing ADE.

A large number of small molecule inhibitors and antibodies (both monoclonal and polyclonal antibodies) have been evaluated for the neutralization of coronaviruses but the results are variable (23, 24, 61–63). Amiodarone is one such small molecule that inhibits SARS infection by interfering with the endocytic pathways (64). However, the clinical efficacy and safety of such small molecules have not been evaluated. The polyclonal antibodies are less well suited for large-scale use and could transmit bound toxic contaminants from other pathogens, allergens and chemicals. Therefore, generating monoclonal antibodies (mAbs) of appropriate specificity against one or the more SARS-CoV2 proteins or even against the host proinflammatory factors is considered. Numerous anti-SARS-CoV2 IgG antibodies against S protein or those targeting RBD have been generated from COVID-19 patients and some have already been characterized. (A compiled list of such antibodies can be found at http://opig.stats.ox.ac.uk/webapps/covabdab/). The primary mechanism of antibody mediated virus neutralization is by blocking the interaction between ACE2 and the RBD either by direct competition or *via* steric hindrance. Interestingly two mAbs, which were originally generated against SARS-CoV S protein, neutralized SARS-CoV2 but did not inhibit ACE2 and RBD interaction (65, 66). The conventional IgG based convalescent therapy or specific IgGs induced during mild infections or even upon vaccination might enhance disease severity through ADE mechanisms (67, 68). ADE is reported for multiple viruses such as dengue viruses (DENV) and respiratory syncytial virus (RSV) and is caused when poorly or non-neutralizing IgGs or other isotypes engage cellular Fc receptors (FcRs) through their fragment constant (Fc) regions (69–71). In so doing such preparations might promote the infectivity of the virus in additional cell types such as monocytes, macrophages, platelets and endothelial cells that are otherwise non-permissive to the infection because of the absence of virus entry receptors. The viruses that mutate frequently or have multiple circulating strains such as DENV and RSV can display ADE phenomenon. Accordingly, the antibodies induced against one strain might not fully neutralize the heterologous strains but can bind to it through fragment antigen-binding (Fab) region, while their Fc regions interact with the FcRs expressed by multiple cell types to facilitate infectivity. Some feline coronaviruses have been shown to exacerbate immunopathology by such extrinsic mechanisms (68, 72, 73) (**Figure 1**). Interestingly, ADE mechanisms could also suppress TLR signaling and reduce the production of type I IFNs by modulating intracellular host factors (74, 75). Therefore, ADE can increase the viral replication and hence produce more virus particles from the infected cells through cell intrinsic mechanisms in addition to increasing the number of infected cell types through extrinsic mechanisms (74, 75). The shorter variants of the conventional full-length antibodies such as the single-chain fragment variable (scFv) and the Fab can preclude the process of ADE but their production in soluble formats generally requires expensive and usually less efficient mammalian expression systems (76, 77).

A potentially interesting approach could involve the generation and use of camelid anti-SARS-CoV2 specific sdAbs (29, 78–81). Such sdAbs are one-tenth the size of conventional antibodies and have superior biological properties suitable for therapy (26, 27). Their extended CDR3 and in some clones CDR2 as well, helps form the projecting paratopes that are suitably poised to neutralize cryptic epitopes that could occur in the fusion peptide or the protease cleavage sites of SARS-CoV2 S protein. Additional features that facilitate the efficient generation of such antibodies include their genetic modifications in the natural host, such as the replacement of hydrophobic amino acids in framework region 2 (FR2) by smaller and hydrophilic residues (V42F, G49Q, L50R, W52G/A) to allow for their expression as the stable and folded products in commonly used microbial species (82). Due to a nucleotide point mutation (G>A) at the conserved 5’ splicing site of the intron, alternative splicing events achieve the deletion of C_H_1 in the mature and processed mRNA to make the association of heavy chains with the light chains impossible (26, 83). Therefore, the libraries of sdAbs can be generated with ease using bacterial, viral, yeast, or ribosomal display approaches (25–28). Moreover, sdAbs are less immunogenic, resist harsh conditions of pH and temperature and can be injected *via* the intranasal route, a desirable attribute for managing respiratory diseases (84). Such sdAbs could be generated for both conformational and linear epitopes of the RBD of SARS-CoV2 S protein to provide an effective and durable blocking as well as hampering the subsequent viral fusion events. In a prefusion complex, RBD undergoes conformational changes from down-conformation (where RBD is in-accessible) to up-conformation (where RBD is accessible). The up-conformation is less stable and interacts with ACE2 (58). During the interaction of RBD and ACE2, the RBD packs into a five-stranded β-sheet structure and acquires a concave shape to fit into the N terminal domain of ACE2. The interaction is stabilized predominantly by hydrophobic interactions (85). As the RBD interacts with ACE2 in up-conformation, the efficient neutralization could be achieved if sdAbs are selected to arrest the RBD in the down-conformation. In the post-fusion complex, the S2 subunit of S protein displays multiple N-linked glycans at Asn1098, Asn1134, Asn1158, Asn1173, and Asn1194. Therefore, sdAbs, which bind to the S2 subunit also need to recognize and interact with the carbohydrate moieties. Other potential targets for selecting sdAbs could be the exposed E and M proteins of SARS-CoV2 as well as the host and viral proteases, as shown for other respiratory infection causing viruses (86–88). The administered anti-SARS-CoV2 sdAbs are not likely to cause an ADE phenomenon as they lack in the Fc region and have minimal to no immunogenicity (**Figure 1**). We, therefore, opine that SARS-CoV2-specific sdAbs represent a better alternative for therapy. Some studies have reported the generation of anti-SARS-CoV2 specific single-domain antibodies and demonstrated their ability to neutralize the virus (A compiled list of such SARS-CoV2 antibodies can be found at http://opig.stats.ox.ac.uk/webapps/covabdab/). The utility of such antibodies in a clinical setting is yet to be shown. SARS-CoV2 specific CD8^+^ T cell responses are primarily directed against ORF1ab and some of the epitopes of S protein. Therefore, such peptides could be fused with sdAbs against the host cell specific surface markers for their efficient delivery to APCs to enhance cross-presentation (30, 89, 90).

## Evolving Managemental Procedures for COVID-19

**Figure 2:**
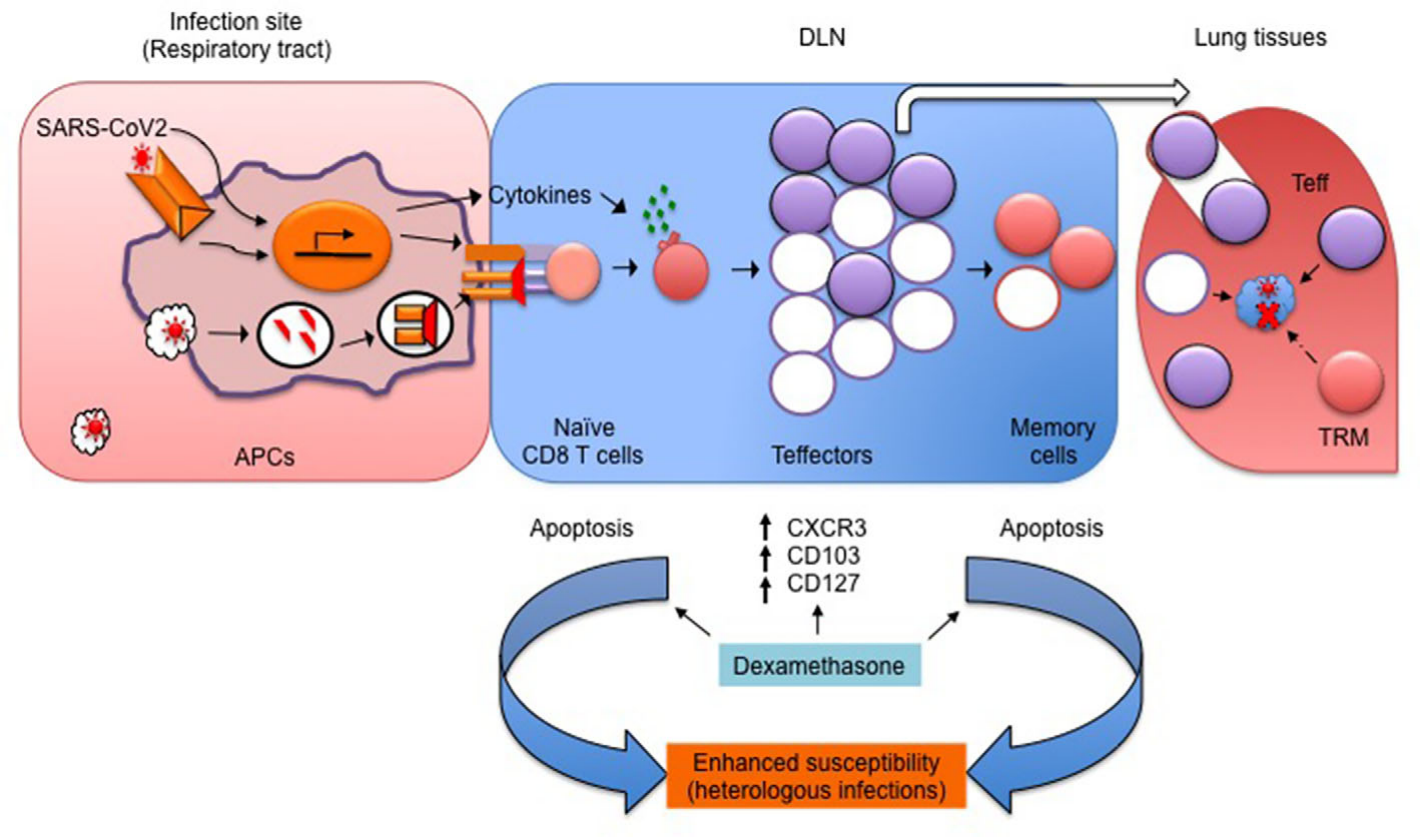
A schematic showing the potential influence of dexamethasone on SARS-CoV2-specific CD8^+^ T cells and its possible ill-effects. SARS-CoV2 derived PAMPs are recognized by APCs, which then process and present viral antigens to generate peptides and activate naïve CD8^+^ T cells. The expanded virus-specific CD8^+^ T cells cytolyze virus infected cells and control virus loads. In the presence of dexamethasone, the activated CD8^+^ T cells (filled purple circles) upregulate molecules such as CD103, CXCR3, and CD127 to facilitate their transport to the infected lung tissues to achieve efficient virus control. A fraction of such cell further differentiates into tissue resident memory (TRM) cells (filled brown circles in DLN/lungs). TRM provide quick protection upon secondary homologous infection. However, dexamethasone, by preferentially killing naïve and memory CD8^+^ T cells, could enhance the host susceptibility to heterologous infections and malignancies.

Patients with severe clinical disease often need oxygen supplementation and mechanical ventilation to prevent hypoxemia and acute respiratory distress syndrome (ARDS) (91, 92). The patients are routinely prescribed antipyretics, supportive fluid therapy and anti-inflammatory drugs. Effective control of the virus is invariably required for a better prognosis, but few if any effective anti-viral modalities are currently available. Coronaviruses, including SARS-CoV2, block interferon response to modulate the innate immunity (46, 47, 93). Therefore, type I IFN therapy, if initiated early in the infection, could reduce virus loads but the associated risks need to be carefully evaluated (49). The sequestration of SARS-CoV2 components that impede type I IFN responses might serve as a potential modality (93). COVID-19 patients administered with IFNα2b showed beneficial effects in controlling their virus loads and had lesser pulmonary damage (94, 95). However, it should be noted that these are only preliminary reports and hence a more stringent, placebo-controlled randomized trial should be conducted before their use in treating COVID-19 patients. (More information on undergoing Phase I clinical trial on safety and efficacy of IFNα2b can be found at https://clinicaltrials.gov/ct2/show/NCT04293887). If the patients are suspected of septicemia as a secondary complication, antibiotics are administered. Many such regimens could induce hyperinflammatory response triggered by bacterial toxins. Therefore, neutralization of endotoxins or exotoxins could have beneficial effects. Such reagents are not currently available for use (94). Immunosuppressive corticosteroid therapy is routinely used to dampen inflammation, but there are conflicting reports on their utility during viral infections. The patients infected with previously circulating SARS-CoV exhibited pulmonary tissue damage, followed by a prolonged recovery phase due to severe lung pathologies but COVID-19 patients, unlike those infected with previously circulating coronaviruses, shed more virus during early stages of infection (85, 95). Therefore, restoring lung function by dampening inflammation was considered to improve disease prognosis. The initial clinical trials showed some beneficial effects of corticosteroids in managing severe cases of COVID-19 (32, 96). The results of a recently concluded larger clinical trial showed a massive survival advantage of ~35% in the severe cases of COVID-19 but to a lesser extent in the milder cases (9). Corticosteroids are potent immunosuppressants and exhibit pleiotropic effects on almost all the organs and cell types (97). However, their effects on anti-viral CD8^+^ T cells recruited during the course of viral infections have not been fully evaluated. We recently demonstrated in a mouse model of herpesvirus infections that dexamethasone exposed naïve and memory CD8^+^ T cells exhibited enhanced susceptibility to apoptosis as compared to their activated counterparts (98). These observations alerted us on their potential long-term implications. For example, the host might become more susceptible to heterologous infections and malignancies due to dexamethasone-induced attrition of naïve CD8^+^ T cells (98). Since the average age of patients administered with dexamethasone in the COVID-19 trial was 66.1 years, such patients are likely to have hematopoietic insufficiencies (42). The preformed memory cells also exhibited enhanced apoptosis upon dexamethasone exposure (98). Therefore, dexamethasone injected patients might lose their immunological memory of the previously encountered pathogens or vaccines. This could further enhance their propensity to acquire such infections. Counterintuitively, the differentiating virus-specific effector CD8^+^ T cells in the presence of dexamethasone upregulated molecules such as CD103 and CXCR3. These molecules promote cellular migration to the infected tissues enriched in IP10 or CXCL10, a ligand for CXCR3 (98, 99). That the treatment of T cells with dexamethasone causes the upregulation of IL7R, a molecule associated with memory phenotype in antigen-stimulated cells, supports the notion that glucocorticoids could additionally promote memory transition (100). Therefore, differentiating virus-specific CD8^+^ T cells could help reduce the virus burden at the infected tissues and additionally acquire a differentiation program to become tissue-resident memory cells (T_RM_). Such cells provide rapid anti-viral effects at tissue sites during secondary homologous infections (**Figure 2**). Some of our unpublished observations support such a hypothesis but extensive analysis at this time is lacking. Therefore, the follow-up studies in COVID-19 patients receiving dexamethasone therapy should aim to investigate such issues in addition to measuring their susceptibility to heterologous infections and cancers.

The anti-inflammatory approaches that have not yet been explored in managing COVID-19 include promoting the endogenous regulatory mechanisms and inhibiting the migration of inflammatory cells to tissue sites. Such strategies could be valuable in the later phase of infection or when the virus is controlled. Chemokine receptor blockade by antibodies or the pharmacological antagonism of sphingosine 1 phosphate receptors with drugs such as fingolimod (FTY720) might be valuable to limit the infiltration of inflammatory cells such to lung tissues (101–103). Furthermore, approaches that can enhance the activity of regulatory cells could help diminish inflammatory response (104). Many approaches have been evaluated in preclinical studies to dampen inflammation by promoting Tregs and these include the infusion of cytokines (IL-2-anti-IL-2 complexes, IL-10, TGF-β), other host-derived molecules such as galectins, neuropeptides, hormones, drugs such as rapamycin (105). None of these approaches have been used for managing COVID-19 cases.

Finally, while many investigators are racing to develop and produce reagents such as antibodies that can reduce the impact of COVID-19, one only hopes that once produced, these remain affordable and unequivocally efficacious.

## Data Availability Statement

The original contributions presented in the study are included in the article/**Supplementary Material**. Further inquiries can be directed to the corresponding author.

## Author Contributions

AD, SD and SS compiled the information. SS and BR presented it logically. All authors contributed to the article and approved the submitted version.

## Funding

The work in the lab of SS is supported by Intramural funding from IISERM and the extramural grant from DBT (BT/PR20283/BBE/117/258/2016).

## Conflict of Interest

The authors declare that the research was conducted in the absence of any commercial or financial relationships that could be construed as a potential conflict of interest.

## Supplementary Material

Supplementary Figure 1 A description of the key events in SARS-CoV entry, replication, and release with potential anti-viral targets. The virus *via* its S glycoprotein interacts with one of known host cell receptor ACE2 and is endocytosed. In the endosome, the spike protein is cleaved by the proteases to initiate further events in viral disassembly and the release of its RNA genome. The copying of RNA genome and assembly of the virus then initiates the release of virus. The potential targets of host immune cells or those achieved by blocking the virus entry and subsequent trafficking events are shown. The molecules that can block the virus entry in the cells either as the soluble mimetics of ACE2 receptor, blocking antibodies against S glycoproteins, host proteins able to bind sugar moieties on the spike proteins can inhibit the viral interaction with membrane expressed ACE2. The virus upon internalization is present in the endosomes. The viral proteins processed and displayed as peptides in context with class I MHC molecules on the surface of infected cells are recognized by effector cytotoxic T cells and such interactions help cytolyze viral infected cells. With the demise of infected cells, the virus burden is controlled. Inhibitors of Papain like protease (PLpro) and Main protease (Mpro) are likely to interfere with the formation of non-structural proteins to interfere with the viral replication in the infected cells. Anti-sense RNAs against viral genome can regulate the synthesis of viral proteins responsible for viral assembly. Question marks indicate lack of detailed information.
