# Supplementary figures and images for: Perspective: Reducing SARS-CoV2 Infectivity and Its Associated Immunopathology
Source: Front Immunol. 2020 Oct 22;11:581076. doi: 10.3389/fimmu.2020.581076 (PMC7642257; doi:10.3389/fimmu.2020.581076)

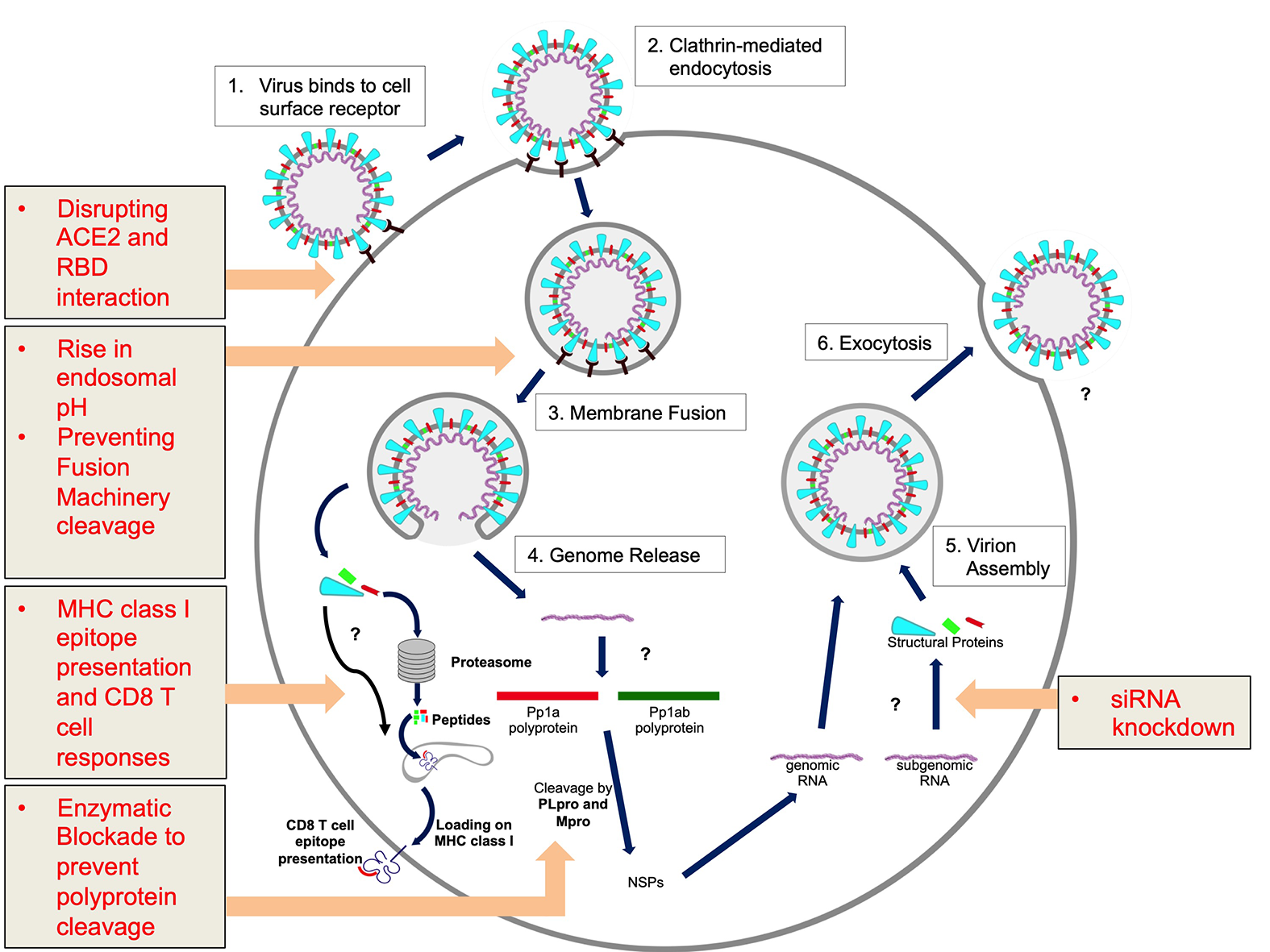

Supplement: Supplementary Figure 1 — A description of the key events in SARS-CoV entry, replication, and release with potential anti-viral targets. The virus via its S glycoprotein interacts with one of known host cell receptor ACE2 and is endocytosed. In the endosome, the spike protein is cleaved by the proteases to initiate further events in viral disassembly and the release of its RNA genome. The copying of RNA genome and assembly of the virus then initiates the release of virus. The potential targets of host immune cells or those achieved by blocking the virus entry and subsequent trafficking events are shown. The molecules that can block the virus entry in the cells either as the soluble mimetics of ACE2 receptor, blocking antibodies against S glycoproteins, host proteins able to bind sugar moieties on the spike proteins can inhibit the viral interaction with membrane expressed ACE2. The virus upon internalization is present in the endosomes. The viral proteins processed and displayed as peptides in context with class I MHC molecules on the surface of infected cells are recognized by effector cytotoxic T cells and such interactions help cytolyze viral infected cells. With the demise of infected cells, the virus burden is controlled. Inhibitors of Papain like protease (PLpro) and Main protease (Mpro) are likely to interfere with the formation of non-structural proteins to interfere with the viral replication in the infected cells. Anti-sense RNAs against viral genome can regulate the synthesis of viral proteins responsible for viral assembly. Question marks indicate lack of detailed information. [file Image_1.tif]
